# Interfacial waveforms in chiral lattices with gyroscopic spinners

**DOI:** 10.1098/rspa.2018.0132

**Published:** 2018-07-25

**Authors:** M. Garau, G. Carta, M. J. Nieves, I. S. Jones, N. V. Movchan, A. B. Movchan

**Affiliations:** 1 School of Computing and Mathematics, Keele University, Keele ST5 5BG, UK; 2 Mechanical Engineering and Materials Research Centre, Liverpool John Moores University, Liverpool L3 3AF, UK; 3 Department of Mechanical, Chemical and Material Engineering, University of Cagliari, Cagliari 09123, Italy; 4 Department of Mathematical Sciences, University of Liverpool, Liverpool L69 7ZL, UK

**Keywords:** elastic lattices, gyroscopic spinners, chiral systems, dispersion, uni-directionalwaveforms

## Abstract

We demonstrate a new method of achieving topologically protected states in an elastic hexagonal system of trusses by attaching gyroscopic spinners, which bring chirality to the system. Dispersive features of this medium are investigated in detail, and it is shown that one can manipulate the locations of stop-bands and Dirac points by tuning the parameters of the spinners. We show that, in the proximity of such points, uni-directional interfacial waveforms can be created in an inhomogeneous lattice and the direction of such waveforms can be controlled. The effect of inserting additional soft internal links into the system, which is thus transformed into a heterogeneous triangular lattice, is also investigated, as the hexagonal lattice represents the limit case of the heterogeneous triangular lattice with soft links. This work introduces a new perspective in the design of periodic media possessing non-trivial topological features.

## 1. Introduction

Systems supporting topologically protected edge modes have attracted increasing attention in recent years. In such systems, which are collectively known as ‘topological insulators’, waves propagating along the edges of the domain are not scattered backwards or inside the medium, even in the presence of imperfections or discontinuities (such as sharp corners and localized defects). Topological insulators are generally characterized by a non-trivial topology of the band structure, associated with the presence of Dirac points at the corners of the Brillouin zone.

The existence of edge modes propagating in one preferential direction was firstly predicted and observed in photonic crystals [1–8], in analogy with electronic edge states in systems exhibiting the quantum Hall effect [9]. Uni-directional topologically protected interfacial states have been identified in [10] for photonic crystals composed of two regions, each containing hexagonally arranged dielectric rods with different radii distributed on a triangular lattice. The effect of singular points on the boundaries of solids through which light propagates has been modelled in [11] for the purpose of developing broadband energy harvesting techniques. Analogues of topological insulators have also appeared in the study of optical lattices [12]. In plasmonics, surface modes were ‘mimicked’ by introducing structured inhomogeneities into the surface [13]. Periodic honeycomb plasmonics capable of supporting uni-directional edge states have appeared in [14,15].

In the context of classical mechanics, there are only a few examples where edge states in structured media have been observed. Acoustic metamaterials supporting topologically protected sound modes have been designed by introducing circulating fluids into a lattice structure [16–19] or by creating interfaces between two phononic crystals having different geometrical properties [20]. An array of acoustic resonators periodically distributed within a hexagonal lattice has been used in [21] to study the robustness of topological edge wave propagation when various defects are embedded in the lattice. The analysis of waves trapped along coastlines fitted with structured barriers has been presented in [22], while similar trapping phenomena have been analysed with an asymptotic model for stratified fluids in [23]. Interfacial waves have been observed in elastic metamaterials, consisting of two slabs with arrangements of defects at two different scales [24]. Topologically protected plates have been constructed by attaching a hexagonal array of resonators possessing different masses to the plate in order to break the inversion symmetry [25]. Preferential directionality has been generated in lattice structures by modifying the tension of the springs connecting the particles [26] or by locally changing the arrangement of masses at the junctions within the structure [27]. Localized interfacial modes for circular arrays of inclusions in membranes have been studied in [28]. The analogue of the quantum Hall effect for mechanical systems has been analytically and experimentally investigated in [29], where topologically protected edge modes have been realized for finite lattice systems whose nodal points were connected to a system of coupled pendula. This has been further explored in [30], where a review of recent attempts in bridging the gap between quantum and classical mechanics for the purpose of designing topological metamaterials has been presented.

In this paper, we consider elastic waves and design an elastic system composed of fundamental mechanical elements capable of supporting and controlling interfacial waveforms. In particular, we consider a hexagonal array of masses connected to gyroscopic spinners at the junctions. Gyroscopic spinners are employed to break the time-reversal symmetry and alter the topology of the band-gaps in correspondence with the Dirac points. We show how interfacial waves propagating in one preferential direction can be generated by dividing the domain of the lattice in two regions, where the spinners rotate in opposite directions. We also demonstrate that the preferential direction can be inverted not only by reversing the direction of the spinners in separate regions, but also by changing the frequency of the excitation applied to a node along the interface.

The first model of an elastic gyro-system was proposed in [31], where both a monatomic and a biatomic lattice were analysed. The dispersion properties of a monatomic lattice with gyroscopic spinners were discussed in more detail in [32], where wave polarization and standing waves were also investigated. It was demonstrated in [33] that in a gyro-lattice with two types of spinners, waves produced by a time-harmonic force with a specific frequency propagate along a single line, which can be diverted by changing the arrangement of the spinners within the medium.

The approach of [31] was employed in [34] to create topologically non-trivial edge waves, whose existence was demonstrated by numerical simulations in the transient regime. In the present paper, attention is focused on interfacial waveforms in a different lattice structure, for which it is possible to derive an analytical expression for the dispersion relation; the simulations are carried out in the time-harmonic regime. Edge waves propagating in one direction in a gyroscopic metamaterial were observed experimentally in [35].

Gyroscopic spinners confer a chiral nature to the system. ‘Chirality’ is the property of an object of not being superimposable onto its mirror image [36]. Chirality has been employed in elastic lattices to create an effective auxetic medium [37,38], to alter the dispersive properties of a system [39,40] and to generate negative refraction [41,42]. Chirality can also be implemented in continuous structural elements, including beams and plates, by using the concept of ‘distributed gyricity’. In particular, the theory of gyro-elastic beams (or ‘gyrobeams’) was proposed in [43] and developed in [44–48], where special attention was given to the study of the eigenfrequencies and eigenmodes of these structural elements. Additionally, in [49] it was shown that gyrobeams can be used as an efficient tool to reduce the vibrations of a structural system in the low-frequency regime.

The paper is organized as follows. In §2, we present a hexagonal chiral system, for which we derive the equations of motion (see §2a) and analyse the dispersion properties (see §2b). In §2c, we demonstrate how to generate interfacial waveforms in this medium by exploiting its topologically non-trivial band structure. In §3, we describe the more general case of a triangular gyro-lattice with links of different stiffness, of which the hexagonal chiral lattice in §2 represents a limit case. After discussing the governing equations and the band diagrams of this system in §3a,b, respectively, we demonstrate that interfacial waveforms with preferential directionality can also be realized in this type of lattice, as detailed in §3c. Finally, in §4 we provide some concluding remarks.

## 2. Hexagonal chiral lattice

**Figure 1.**
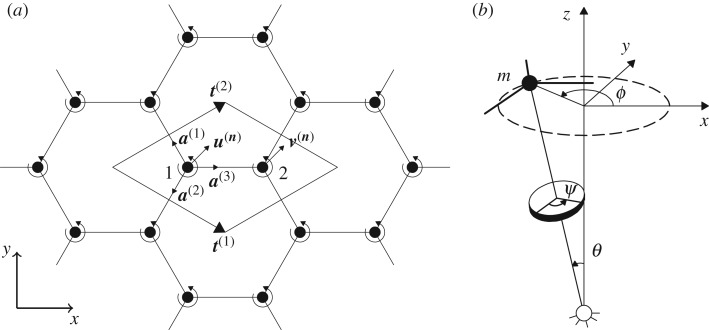
(*a*) Hexagonal lattice connected to a uniform system of gyroscopic spinners, where ***u***^(***n***)^ and ***v***^(***n***)^ denote the displacements of the two lattice particles of the elementary cell; (*b*) representation of a gyroscopic spinner, where *ψ*, *ϕ* and *θ* are the angles of spin, precession and nutation, respectively.

We consider a hexagonal lattice of masses *m*, connected by non-inertial elastic links of length *l* and stiffness *c*, as shown in figure 1*a*. The masses of the lattice are attached to gyroscopic spinners, which are geometrically identical and spin with the same rate and in the same direction. Each spinner is hinged at the bottom end, allowing rotations but not translations. At the top end, it undergoes the same displacement as the lattice particle to which it is connected. A schematic representation of each gyroscopic spinner is illustrated in figure 1*b*, where the angles *ψ*, *ϕ* and *θ* are the angles of spin, precession and nutation, respectively.

In the undeformed configuration, the axis of each spinner is perpendicular to the *xy*-plane. When the lattice masses move due to an incoming wave, the spinners start precessing and exert a force that is perpendicular to the mass displacement. Here we assume that *θ* is small and gravity forces are negligible, so that the lattice particles are constrained to move in the *xy*-plane.

### (a) Governing equations

The vectors $t^{\left(1\right)}={\left(3l/2,-\sqrt{3}l/2\right)}^{T}$ and $t^{\left(2\right)}={\left(3l/2,\sqrt{3}l/2\right)}^{T}$, indicated in figure 1*a*, define the periodicity of the system. The position of each lattice particle is given by ***x***^(***n***)^ = ***x***^(**0**)^ + *n*_1_***t***^(1)^ + *n*_2_***t***^(2)^, where ***n*** = (*n*_1_, *n*_2_)^T^ is the multi-index and ***x***^(**0**)^ is the position of a reference particle in the lattice.

The elementary cell of the periodic structure consists of two particles, whose displacements are denoted by ***u***^(***n***)^ and ***v***^(***n***)^ (figure 1*a*). The equations of motion of the two lattice particles in the time-harmonic regime are given by

2.1*a* $$-m\omega^{2}u^{\left(n\right)}=c\sum_{j=1}^{3}\;[a^{\left(j\right)}\cdot (v^{\left(n-e_{j}\right)}-u^{\left(n\right)})]a^{\left(j\right)}+i\alpha \omega^{2}Ru^{\left(n\right)}$$

and

2.1*b* $$-m\omega^{2}v^{\left(n\right)}=c\sum_{j=1}^{3}\;[a^{\left(j\right)}\cdot (u^{\left(n+e_{j}\right)}-v^{\left(n\right)})]a^{\left(j\right)}+i\alpha \omega^{2}Rv^{\left(n\right)},$$

where *ω* is the radian frequency, and the vectors ***e***^(1)^ = (1, 0)^T^, ***e***^(2)^ = (0, 1)^T^ and ***e***^(3)^ = (0, 0)^T^ are used to specify the positions of the neighbouring particles. The unit vectors ***a***^(*j*)^ in (2a) define the directions of the lattice links (figure 1*a*):

2.2 $$a^{\left(1\right)}={\left(-\frac{1}{2},\frac{\sqrt{3}}{2}\right)}^{T},\;a^{\left(2\right)}={\left(-\frac{1}{2},-\frac{\sqrt{3}}{2}\right)}^{T}\;\mathrm{and}\;a^{\left(3\right)}={\left(1,0\right)}^{T},$$

while the matrix ***R*** is the rotation matrix

2.3 $$R=\left(\begin{array}{cc} 0 & 1\\ -1 & 0 \end{array}\right).$$

The parameter *α* in (2a) represents the spinner constant, which was obtained in [31] to satisfy the compatibility of the moving spinner and vibrating time-harmonic lattice. In this framework, the small nutation angle *θ* is time-harmonic and its radian frequency *ω* is equal to the radian frequency of the vibrating lattice. The spinner constant *α* depends on the geometry of the spinners [31], as shown in the derivation of this constant in the electronic supplementary material.

The quasi-periodicity of the system is described by the Bloch–Floquet conditions:

2.4 $$W(r+n_{1}t^{\left(1\right)}+n_{2}t^{\left(2\right)})=W(r)\;e^{ik\cdot Tn},$$

where ***W*** = (*u*_*x*_, *u*_*y*_, *v*_*x*_, *v*_*y*_)^T^ is the displacement vector, ***r*** = (*x*, *y*)^T^ is the position vector, ***k*** = (*k*_1_, *k*_2_)^T^ is the wavevector (or Bloch vector) and

2.5 $$T=(t^{\left(1\right)},t^{\left(2\right)})=l\;\left(\begin{array}{ll} \frac{3}{2} & \frac{3}{2}\\ -\frac{\sqrt{3}}{2} & \frac{\sqrt{3}}{2} \end{array}\right).$$

By introducing (2.4) into (2a), we obtain the following system of equations in matrix form:

2.6 $$[C-\omega^{2}(M-A)]W=0,$$

where ***M*** = *m****I*** is the mass matrix (***I*** is the 4 × 4 identity matrix),

2.7 $$A=i\alpha \left(\begin{array}{cccc} 0 & -1 & 0 & 0\\ 1 & 0 & 0 & 0\\ 0 & 0 & 0 & -1\\ 0 & 0 & 1 & 0 \end{array}\right)$$

is the spinner matrix, and

2.8 $$C=c\;\left(\begin{array}{cccc} \frac{3}{2} & 0 & -1-\frac{{\text{e}}^{-\text{i}\eta }+{\text{e}}^{-\text{i}\gamma }}{4} & \frac{\sqrt{3}({\text{e}}^{-\text{i}\eta }-{\text{e}}^{-\text{i}\gamma })}{4}\\ 0 & \frac{3}{2} & \frac{\sqrt{3}({\text{e}}^{-\text{i}\eta }-{\text{e}}^{-\text{i}\gamma })}{4} & -\frac{3({\text{e}}^{-\text{i}\eta }+{\text{e}}^{-\text{i}\gamma })}{4}\\ -1-\frac{{\text{e}}^{\text{i}\eta }+{\text{e}}^{\text{i}\gamma }}{4} & \frac{\sqrt{3}({\text{e}}^{\text{i}\eta }-{\text{e}}^{\text{i}\gamma })}{4} & \frac{3}{2} & 0\\ \frac{\sqrt{3}({\text{e}}^{\text{i}\eta }-{\text{e}}^{\text{i}\gamma })}{4} & -\frac{3({\text{e}}^{\text{i}\eta }+{\text{e}}^{\text{i}\gamma })}{4} & 0 & \frac{3}{2} \end{array}\right)$$

is the stiffness matrix, where $\eta =(3k_{1}-\sqrt{3}k_{2})l/2$ and $\gamma =(3k_{1}+\sqrt{3}k_{2})l/2$. We note that the determinant of ***C*** is zero for any value of the Bloch vector. This implies that the system is statically under-constrained (see also [50]). However, in the dynamic case the inertial forces can balance external loads. Typically, static honeycomb structures are designed as frames with rigid connections at the junctions [51]. Here, we have considered pin joints in order to solve the problem semi-analytically.

### (b) Dispersion properties

For non-trivial solutions of (2.6) to exist, the following equation must hold:

2.9 $$\det[C-\omega^{2}(M-A)]=0,$$

which represents the dispersion relation of the chiral system considered. Equation (2.9) is an algebraic equation of fourth order in *ω*^2^ and it embeds the action due the gyroscopic motion of the spinners through the matrix ***A***.

We normalize (2.9) by introducing the non-dimensional scalar quantities $\tilde{\alpha }=\alpha /m$, $\tilde{\omega }=\omega \sqrt{m/c}$ and $\tilde{k}=kl$ and the non-dimensional matrices ***C̃*** = ***C***/*c* and ***Ã*** = ***A***/*m*. This leads to a normalized form of the dispersion relation:

2.10 $$\det[\tilde{C}-{\tilde{\omega }}^{2}(I-\tilde{A})]=0.$$

In the following, the symbol ‘tilde’ will be omitted for ease of notation.

One solution of (2.9) is *ω* = 0 for any value of the wavevector ***k***. This solution represents a rigid-body motion of the system, which is statically under-constrained (see also [50]). In §3, it is shown that rigid-body motions are prevented if internal links are introduced, converting the hexagonal lattice into a heterogeneous triangular lattice.

When *α* < 1, (2.9) admits three real and positive solutions in *ω* (in addition to *ω* = 0), which yield three dispersion surfaces. When *α* > 1, two solutions for *ω*^2^ are negative and hence only one dispersion surface is present.

**Figure 2.**
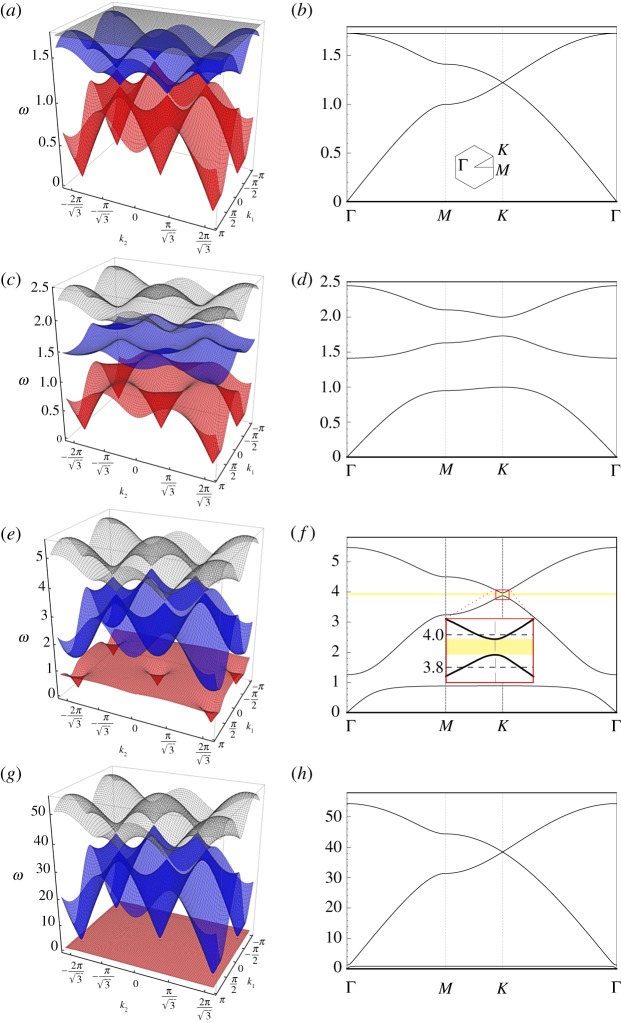
Dispersion surfaces (*a*,*c*,*e*,*g*) and corresponding cross-sections (*b*,*d*,*f* ,*h*), determined for *α* = 0 (*a*,*b*), *α* = 0.5 (*c*,*d*), *α* = 0.9 (*e*,*f* ), *α* = 0.999 (*g*,*h*). For *α* < 1, the discrete hexagonal system exhibits three dispersion surfaces; the fourth solution of the dispersion relation, given by *ω* = 0, is not shown in the figures for the sake of clarity. Note that the scales of the vertical axes are different, since the dispersion surfaces move to higher frequencies as *α* → 1. (Online version in colour.)

Figure 2 shows the dispersion surfaces of the chiral hexagonal lattice for different values of the spinner constant *α*. The diagrams on the right represent the dispersion diagrams, calculated along the path Γ*MK*Γ in the reciprocal lattice, shown in the inset of figure 2*b*. The coordinates of the points Γ, *M* and *K* are the following: Γ = (0, 0), *M* = (2*π*/(3*l*), 0), $K=(2\pi /(3l),2\pi /(3\sqrt{3}l))$. The vectors ***b***^(1)^ and ***b***^(2)^, describing the periodicity of the reciprocal lattice, are given by

2.11 $$b^{\left(1\right)}=2\pi \frac{\tau^{\left(2\right)}\times \kappa }{\tau^{\left(1\right)}\cdot (\tau^{\left(2\right)}\times \kappa )}=\left(\begin{array}{c} \frac{2\pi }{3l}\\ -\frac{2\pi }{\sqrt{3}l}\\ 0 \end{array}\right)\;\;\text{and}\;b^{\left(2\right)}=2\pi \frac{\kappa \times \tau^{\left(1\right)}}{\tau^{\left(1\right)}\cdot (\tau^{\left(2\right)}\times \kappa )}=\;\left(\begin{array}{c} \frac{2\pi }{3l}\\ \frac{2\pi }{\sqrt{3}l}\\ 0 \end{array}\right),$$

where ***τ***^(*j*)^ = ((***t***^(*j*)^)^T^, 0)^T^, *j* = 1, 2, and ***κ*** is the unit vector perpendicular to the lattice plane and directed along the positive *z*-axis.

When there are no spinners attached to the lattice (*α* = 0), one solution is $\omega =\sqrt{3}$ for any value of the wavevector ***k***. The other two non-trivial solutions describe dispersion surfaces that intersect at a Dirac point, whose frequency is $\omega =\sqrt{\frac{3}{2}}$. We note that the dispersion diagrams in figure 2*a*,*b* for *α* = 0 are in agreement with [52]. The appearance of the Dirac points has been suggested to be linked to hidden symmetries [53] and mirror symmetries [54] of the medium.

When 0 < *α* < 1, the lowest nonzero dispersion surface decreases as *α* is increased, while the highest two dispersion surfaces increase. As a consequence, two finite stop-bands appear within the band diagram of the gyro-lattice. Furthermore, the highest dispersion surface is no longer ***k***-independent as in the case of *α* = 0. The width of the upper finite stop-band tends to zero as *α* → 1^−^, and a new Dirac point appears at a frequency larger than that for the case of *α* = 0. Figure 2*g*,*h* shows the dispersion diagrams for *α* = 0.999; in this case, it is apparent that a new Dirac point is forming, and that the lowest non-zero dispersion surface is almost flat, except in a small proximity of point Γ.

**Figure 3.**
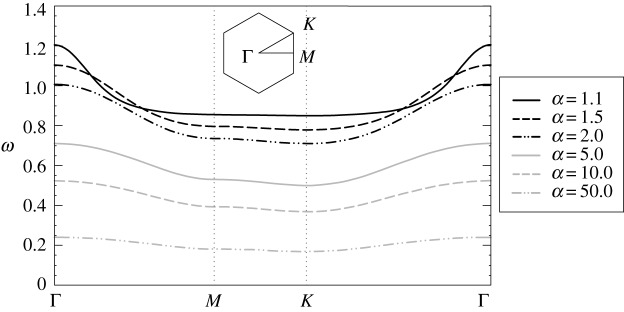
Dispersion diagrams for the hexagonal chiral lattice, computed for values of *α* > 1. The discrete hexagonal system is characterized by only one dispersion surface when *α* > 1; another real solution of the dispersion relation is *ω* = 0 for any wavevector ***k***.

When *α* > 1, there is only one dispersion surface, apart from *ω* = 0. A similar phenomenon was observed for the triangular lattice with gyroscopic spinners [31,32], where there is only one dispersion surface in the supercritical regime *α* > 1. In contrast to the triangular lattice, the dispersion surface of the hexagonal lattice, for *α* > 1, does not pass through the origin. Figure 3 shows that the dispersion surface decreases and becomes flatter as the spinner constant increases; in the limit when *α* → ∞, it tends to zero for any wavevector ***k***.

### (c) Excitations leading to waveforms with preferential directionality

In this section, we show the results of numerical simulations, where a time-harmonic displacement is applied to a particle in the hexagonal chiral lattice. The imposed displacement is ***U***_0_e^−i*ωt*^, where |***U***_0_| is the amplitude and *t* is time. The gyroscopic spinners are used to break the time-reversal symmetry and hence waves are expected to propagate along a preferential direction.

The time-harmonic response of the chiral lattice is analysed using Comsol Multiphysics (v. 5.2a). The lattice is modelled as a system of massless rods connected by hinges, and point masses are incorporated at the junctions. In the finite-element computations, the gyroscopic effect is taken into account by introducing a force proportional to the particle displacement at each lattice junction (see the last terms of equations (2a)). In order to prevent rigid-body motions of the whole system, the displacements of the masses at the corners of the model are set equal to zero. In addition, perfectly matched layers (PML) are attached to the sides of the model to avoid reflections of waves generated by the time-harmonic displacement. In this way, the system is modelled as a spatially infinite medium. PML are created by using viscous dampers, whose parameters are tuned to minimize the reflection coefficient [32,55].

We define the cell containing the point with the imposed displacement by the multi-index n* = (n*_1_, n*_2_)^T^. In this cell, we apply the condition

2.12 $$\delta_{j1}u^{\left(n^{*}\right)}+\delta_{j2}v^{\left(n^{*}\right)}=U_{0},$$

where *δ*_*ij*_ is the Kronecker delta and *j* = 1 or 2 will correspond to a time-harmonic displacement applied to the node labelled 1 or 2, respectively, in the cell of figure 1*a*. The equations of motion for the nodes without imposed displacement are given by (2a).

In what follows, we show that localized interfacial waveforms can be supported in an inhomogeneous hexagonal lattice attached to gyroscopic spinners. In particular, the inhomogeneity of the lattice is brought by inserting gyroscopic spinners having different spinner constants, which are chosen equal to ± 0.9 in all examples considered. In addition, we demonstrate that the direction of these waveforms can be controlled by either altering the spin directions of the gyroscopic spinners or changing the frequency of the applied displacement.

In the first set of simulations, we divide the lattice domain into two different regions. The regions are distinguished by having gyroscopic spinners characterized by spinner constants of the same magnitude but of opposite sign. At specific frequencies, which can be predicted from the dispersion analysis described in §2b, interfacial waves propagate along the internal boundaries between the two regions. In these computations, the lattice is a 150*l* × 110*l* rectangle.

**Figure 4.**
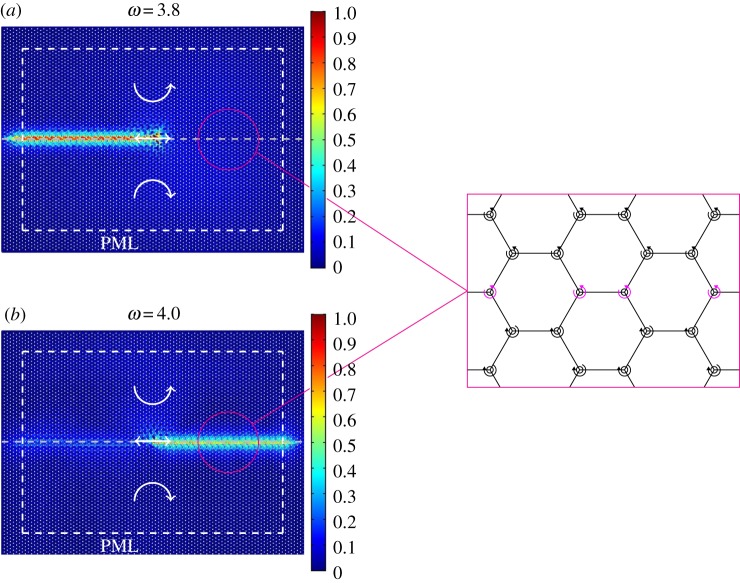
Relative displacement amplitude fields in the chiral lattice at different frequencies of the applied time-harmonic displacement and for different spin directions of the spinners (indicated by the white circular arrows) above and below the interface. The time-harmonic displacement (represented by a straight white arrow) is applied to the central node of the lattice. In these computations, the absolute value of the spinner constant is |*α*| = 0.9, and the frequency of the applied displacement is (*a*) *ω* = 3.8 and (*b*) *ω* = 4.0 (see also figure 2*f* ). PML are attached to the sides of the model, as indicated by the dashed white lines. The interfaces between regions where spinners rotate in opposite directions are represented by dashed grey lines. To the right, we provide a magnification of the interface, where the spinners on the interface are shown in a different colour. The diagrams represent the ratios of the displacement amplitudes to the amplitude of the imposed displacement. (Online version in colour.)

In figure 4*a*,*b*, we divide the lattice domain into two regions separated by a horizontal interface, where the spinners rotate as shown in the close-up view. In the upper and lower regions, gyroscopic spinners have the same spin rate (|*α*| = 0.9) but they spin in opposite directions, as indicated by the white circular arrows. At the central node of the domain, we impose a time-harmonic displacement, whose direction is represented by a white arrow. Figure 4*a* illustrates the displacement field calculated for the applied displacement of the radian frequency *ω* = 3.8, which is below the lower limit of the stop-band highlighted in figure 2*f* , related to the problem concerning free vibrations in the homogeneous system. From figure 4*a*, it is apparent that waves propagate along the interface between the two regions in one direction. It has also been found that the direction of the interfacial wave can be reversed by swapping the spin directions of the spinners in the two regions. Figure 4*b* shows the interfacial waves generated by the applied displacement of the radian frequency *ω* = 4.0. This frequency is above the upper limit of the stop-band highlighted in figure 2*f* . Comparing figure 4*a*,*b*, we observe that when the frequency of the applied displacement is chosen in the proximity of the upper limit of the stop-band in figure 2*f* , waves propagate in the opposite direction compared to the case when the frequency is close to the lower limit of this stop-band.

**Figure 5.**
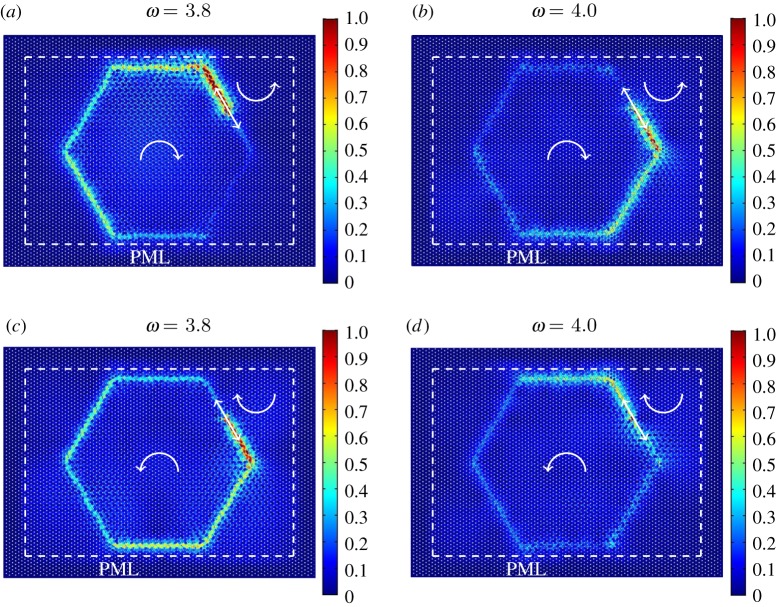
Interfacial waveforms with preferential directionality in a hexagonal lattice connected to a system of gyroscopic spinners. The lattice domain is divided into two regions: in the hexagonal region the spinners rotate (*a*,*b*) clockwise and (*c*,*d*) anticlockwise, while the ambient medium contains spinners which rotate in the opposite direction to those situated inside the hexagonal region. The absolute value of the spinner constant is |*α*| = 0.9 for all the gyroscopic spinners. At the interface, the spinners rotate in the same direction as in the exterior domain. A time-harmonic displacement, represented by the straight arrow, is applied to a node on the interface of these regions. The relative displacement amplitude fields are obtained for the applied displacement of frequency (*a*,*c*) *ω* = 3.8 and (*b*,*d*) *ω* = 4.0 (see also inset in figure 2*f* ). The figures illustrate that the preferential direction of the interfacial waveform can be influenced by interchanging the direction of rotation of the spinners or by changing the frequency of the external excitation. (Online version in colour.)

Another example of an interfacial waveform is presented in figure 5. In this case, the interface has a hexagonal shape. Figure 5*a*,*b* shows the displacement fields computed for two different values of the frequency of the imposed displacement, specified at the top of each figure (see also figure 2*f* ); in both figure 5*a*,*b*, the spinners inside the hexagonal domain rotate clockwise, while the spinners in the ambient medium rotate anticlockwise. The comparison between figure 5*a*,*b* emphasizes the dependence of the preferential directionality of the medium on the frequency of the excitation, which can be predicted from the dispersion analysis discussed in §2b.

In order to better visualize the phenomenon, shown in figure 5*a*,*b*, we illustrate the motion of the particles in the proximity of the point of excitation with two videos, included in the electronic supplementary material. The electronic supplementary material videos show a narrow region of the gyro-elastic lattice, where the dashed red line represents the interface and the blue arrow indicates the direction of the applied displacement. It is clear that in electronic supplementary material video 1, corresponding to the case presented in figure 5*a*, the displacements of the lattice particles are significant in the direction along the dashed red line above the applied displacement, while they are small below the point where this displacement is applied. The direction of propagation is indicated with an arrow. The opposite scenario is observed in electronic supplementary material video 2 related to the configuration of figure 5*b*.

In figure 5*c*,*d*, the spinners inside the hexagonal lattice spin anticlockwise, while those in the ambient medium spin clockwise. Comparing figure 5*c*,*a* and figure 5*d*,*b*, it is apparent that interchanging the directions of spin for the gyroscopes reverses the direction of the interfacial waveform.

**Figure 6.**
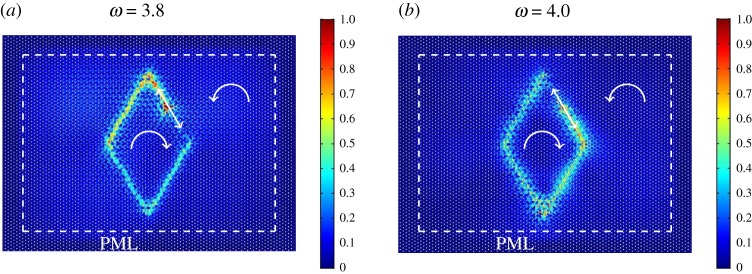
Another example showing the sensitivity of the preferential directionality of an interfacial waveform in a hexagonal lattice on spinners, containing a subdomain where the spinners rotate in the opposite direction to those in the ambient medium. The radian frequency of the applied displacement is (*a*) *ω* = 3.8 and (*b*) *ω* = 4.0 (see also figure 2*f* ). As in the previous simulations, the absolute value of the spinner constant is |*α*| = 0.9 throughout the domain. (Online version in colour.)

The effect of uni-directional localization shown in figure 5 is not unique to the hexagonal subdomain within the inhomogeneous lattice. A final illustration of an interfacial waveform along the boundary of a different subset of an inhomogeneous hexagonal lattice is given in figure 6, where the internal boundary is now a rhombus. In particular, figure 6*a*,*b* are obtained for a radian frequency of the applied displacement equal to *ω* = 3.8 and *ω* = 4.0, respectively. These frequencies are indicated in the magnified inset of figure 2*f* . Once again, by changing the frequency of the external excitation, it is possible to alter the direction of the waveform.

We emphasize that, in all simulations presented in this section, the results are found to be independent of the direction of the external excitation. We also observe that if the spin directions of the spinners on the interface are reversed, the wave pattern is not affected. Finally, it is interesting to note that interfacial waves propagating in the vertical direction were not observed in such a structure.

## 3. A degenerating triangular chiral lattice

The hexagonal lattice described in §2 is statically under-constrained and its dispersion diagram is characterized by a single acoustic branch for *α* < 1 (figure 2). In order to allow for propagation of both shear and pressure waves in the medium, we introduce internal links into the structure, which then becomes a triangular lattice. This lattice degenerates into the hexagonal lattice of §2 when the stiffness of these internal links tends to zero.

**Figure 7.**
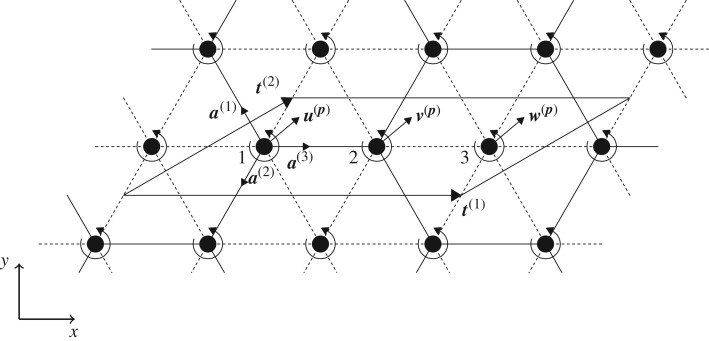
An inhomogeneous triangular lattice structure linked to a system of spinners. The elementary cell for this structure is shown, which contains three particles having displacements ***u***^(***p***)^, ***v***^(***p***)^ and ***w***^(***p***)^. The lattice is composed of springs with stiffnesses *c* (solid lines) and *c*_*ε*_ (dashed lines), where *c*_*ε*_≤*c*. Springs possessing stiffness *c*_*ε*_ form the internal connections of hexagon vertices in the lattice. Here, the lattice basis vectors are taken as ***t***^(1)^ = (3*l*, 0)^T^ and $t^{\left(2\right)}={\left(3l/2,\sqrt{3}l/2\right)}^{T}$.

This triangular lattice is shown in figure 7. It is composed of particles with mass *m* and two types of springs, each possessing length *l* and having stiffnesses *c* and *c*_*ε*_. The stiffness *c*_*ε*_ is assumed to be smaller than or equal to *c*. As before, each mass is connected to a spinner that has a spinner constant *α*.

The elementary cell for this configuration is shown in figure 7, where it can be seen that this cell contains three particles, whose displacements are denoted as ***u***^(***p***)^, ***v***^(***p***)^ and ***w***^(***p***)^. In addition, the basis vectors ***t***^(1)^ = (3*l*, 0)^T^ and $t^{\left(2\right)}={\left(3l/2,\sqrt{3}l/2\right)}^{T}$ are used to define the periodicity of the system. The unit vectors ***a***^(*j*)^, *j* = 1, 2, 3, introduced in (2.2) will also be used to define the directions of the links in the lattice. A particle's position in this lattice can be determined through ***x***^(***p***)^ = ***x***^(**0**)^ + *p*_1_***t***^(1)^ + *p*_2_***t***^(2)^, where ***p*** = (*p*_1_, *p*_2_)^T^ is a multi-index.

### (a) Governing equations of the heterogeneous triangular lattice

In the time-harmonic regime, the governing equations of the three particles in the elementary cell are

3.1*a* $$\begin{array}{rl} -m\omega^{2}u^{\left(p\right)} & =\sum_{j=1}^{3}[a^{\left(j\right)}\cdot (c(v^{\left(p-q_{j}\right)}-u^{\left(p\right)})+c_{\varepsilon }(w^{\left(p+q_{j}-e_{1}\right)}-u^{\left(p\right)}))]a^{\left(j\right)}+i\alpha \omega^{2}Ru^{\left(p\right)}, \end{array}$$

3.1*b* $$\begin{array}{rl} -m\omega^{2}v^{\left(p\right)} & =\sum_{j=1}^{3}[a^{\left(j\right)}\cdot (c(u^{\left(p+q_{j}\right)}-v^{\left(p\right)})+c_{\varepsilon }(w^{\left(p-q_{j}\right)}-v^{\left(p\right)}))]a^{\left(j\right)}+i\alpha \omega^{2}Rv^{\left(p\right)} \end{array}$$

3.1*c* $$\begin{array}{rl} \;\text{and}\;-m\omega^{2}w^{\left(p\right)} & =c_{\varepsilon }\sum_{j=1}^{3}[a^{\left(j\right)}\cdot (v^{\left(p+q_{j}\right)}+u^{\left(p-q_{j}+e_{1}\right)}-2w^{\left(p\right)})]a^{\left(j\right)}+i\alpha \omega^{2}Rw^{\left(p\right)}, \end{array}$$

where ***q***_1_ = ***e***_1_ − ***e***_2_, ***q***_2_ = ***e***_2_, ***q***_3_ = 0***e***_1_ + 0***e***_2_ and ***R*** is the rotation matrix in (2.3). We note that (3.1a,*b*) reduce to (2.1a,*b*) if *c*_*ε*_ = 0, while in this case (3.1c) implies ***w***^(***p***)^ vanishes for *ω* > 0.

We proceed to analyse the Bloch–Floquet modes of the above system by introducing the quasi-periodicity conditions given in (2.4) and (2.5), where ***W*** = (*u*_*x*_, *u*_*y*_, *v*_*x*_, *v*_*y*_, *w*_*x*_, *w*_*y*_)^T^ is the new displacement vector, ***T*** = (***t***^(1)^, ***t***^(2)^) is constructed from the new lattice basis vectors and ***p*** replaces ***n***. In a similar way to that described in §2, we then arrive at a system of equations for ***W*** in the form

3.2 $$[C_{\varepsilon }-\omega^{2}(M-A)]W=0,$$

where ***M*** = *m****I*_6_** (***I*_*j*_** is the *j* × *j* identity matrix), the spinner matrix

3.3 $$A=i\alpha \;\mathrm{diag}\left(\left(\begin{array}{cc} 0 & -1\\ 1 & 0 \end{array}\right),\left(\begin{array}{cc} 0 & -1\\ 1 & 0 \end{array}\right),\left(\begin{array}{cc} 0 & -1\\ 1 & 0 \end{array}\right)\right),$$

and ***C***_*ε*_ is a 6 × 6 stiffness matrix depending on the wavevector ***k*** given by

3.4 $$C_{\varepsilon }=\left(\begin{array}{cc} C_{\varepsilon }^{\left(1\right)} & C_{\varepsilon }^{\left(3\right)}\\ {\left(\bar{C_{\varepsilon }^{\left(3\right)}}\right)}^{\text{T}} & C_{\varepsilon }^{\left(2\right)} \end{array}\right),$$

with

3.5 $$C_{\varepsilon }^{\left(1\right)}=C+\frac{3}{2}c_{\varepsilon }I_{4},\;C_{\varepsilon }^{\left(2\right)}=3c_{\varepsilon }I_{2}$$

and

3.6 $$C_{\varepsilon }^{\left(3\right)}=\frac{c_{\varepsilon }}{4}\left(\begin{array}{cc} -(4e^{-i\mu }+e^{-i\eta }+e^{-i\gamma }) & \sqrt{3}(-e^{-i\eta }+e^{-i\gamma })\\ -\sqrt{3}(e^{-i\eta }-e^{-i\gamma }) & -3(e^{-i\eta }+e^{-i\gamma })\\ -(4+e^{-i\eta }+e^{-i\gamma }) & \sqrt{3}(e^{-i\eta }-e^{-i\gamma })\\ \sqrt{3}(e^{-i\eta }-e^{-i\gamma }) & -3(e^{-i\eta }+e^{-i\gamma }) \end{array}\right).$$

Here $\mu =3k_{1}l,\eta =(3k_{1}-\sqrt{3}k_{2})l/2$, $\gamma =(3k_{1}+\sqrt{3}k_{2})l/2$, and *ε* = *c*_*ε*_/*c*. The bar in (3.4) denotes the complex conjugate.

Non-trivial solutions then follow from the roots of the determinant of the coefficient matrix in (3.2), which yields a polynomial of the sixth order in *ω*^2^. We apply the same normalizations as in §2, except we introduce ***C̃***_*ε*_ = ***C***_*ε*_/*c* (where the ‘tilde’ will again be omitted for convenience in what follows). For *ε* → 0, we analyse the behaviour of eigenfrequencies as solutions associated with

3.7 $$\det[C_{\varepsilon }-\omega^{2}(I-A)]=0.$$

### (b) Dispersive features of the chiral triangular lattice as it degeneratesto a chiral hexagonal lattice

In this section, we discuss the dispersion properties of the inhomogeneous chiral triangular structure. In particular, we determine the dispersion curves for this system in the reciprocal lattice space along the same path ΓMKΓ described in §2b.

**Figure 8.**
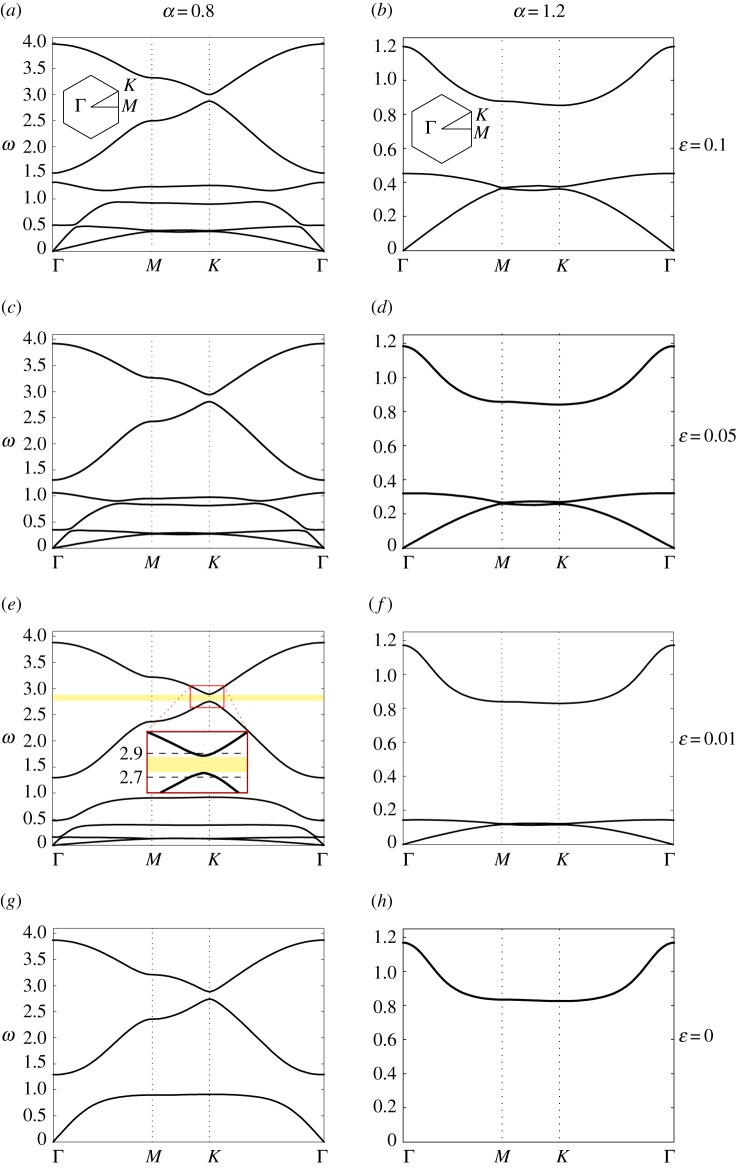
Dispersion diagrams for the chiral triangular lattice, determined for (*a*,*c*,*e*,*g*) *α* = 0.8 and (*b*,*d*,*f* ,*h*) *α* = 1.2 and for decreasing values of *ε*: (*a*,*b*) *ε* = 0.1, (*c*,*d*) *ε* = 0.05, (*e*,*f* ) *ε* = 0.01, (*g*,*h*) *ε* = 0. The discrete triangular system exhibits six (three) dispersion surfaces for *α* < 1 (*α* > 1). A magnified inset of the neighbourhood of point K is included in (*e*) for later use (see §3c). (Online version in colour.)

In figure 8, we present dispersion diagrams based on (3.7). We note that (3.7) does not allow for rigid-body motions in contrast to the hexagonal lattice of §2. When 0≤*α* < 1, relation (3.7) admits six positive solutions for *ω*, namely two acoustic branches and four optical branches. On the other hand, if *α* > 1, only three positive solutions of (3.7) exist (the other three solutions for *ω*^2^ are negative). This can be observed, for instance, in figure 8*a*,*b*, where dispersion curves based on (3.7) have been presented for *α* = 0.8 and *α* = 1.2, respectively; in both figures, the ratio of the stiffnesses of the two types of links is *ε* = 0.1. Therefore, the inhomogeneous triangular lattice exhibits three additional wave modes compared with the hexagonal structure when *α* < 1 and two additional modes when *α* > 1.

For *α* < 1, figure 8*a*,*c*,*e*,*g* shows the dispersion diagrams of the triangular lattice as the stiffness *c*_*ε*_ decreases to zero. In these figures, we observe that when *α* < 1 there are three finite stop-bands. As *ε* decreases, the six dispersion curves move to lower frequencies and the widths of the lowest two finite stop-bands decrease and they disappear when *ε* = 0. In this limit, there are only three dispersion curves in figure 8*g*. These curves represent the non-trivial branches associated with the dispersion relation (2.10) of the hexagonal lattice connected to gyroscopic spinners, described in detail in §2b (see also (3.1a–*c*)).

Similar behaviour is observed for *α* > 1 and *ε* → 0 in figure 8*b*,*d*,*f* ,*h*. In this case, one finds three dispersion curves and two finite stop-bands. All curves move to lower frequencies as *ε* → 0. The lowest two dispersion curves decrease and flatten with decrease of *ε* causing the lowest stop-band to shrink. These two curves approach zero and disappear as *ε* → 0, thus retrieving the case of a hexagonal lattice connected to gyroscopic spinners when *ε* = 0 (figure 8*h*).

**Figure 9.**
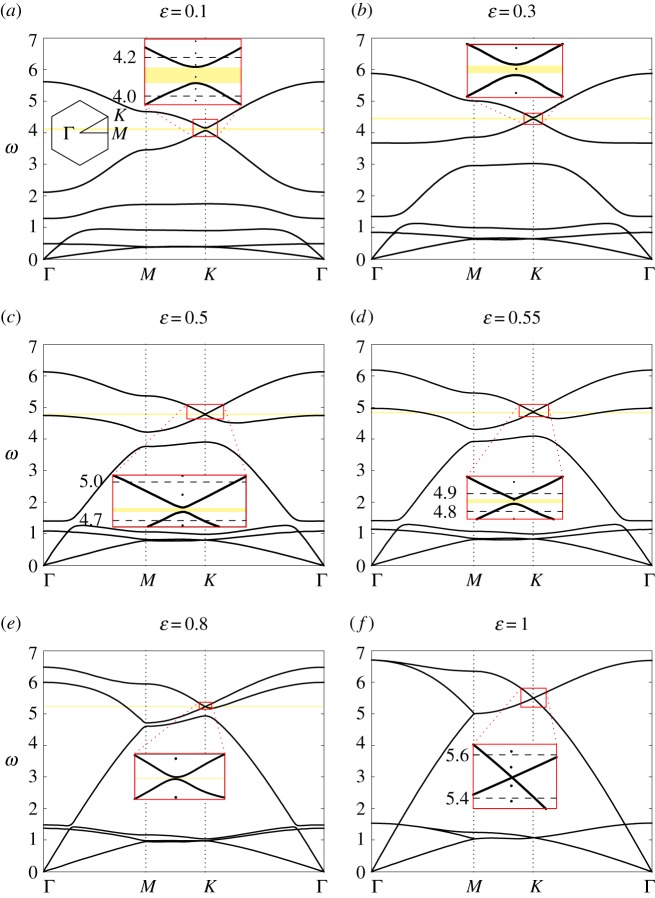
Dispersion diagrams for the chiral triangular lattice, calculated for *α* = 0.9 and for different values of *ε*, as specified. Magnified insets of the neighbourhood of point K are also presented. (Online version in colour.)

Figure 9 shows the dispersion diagrams for the triangular lattice, determined for a different value of the spinner constant, *α* = 0.9, and larger values for the stiffness of the internal links, proportional to *ε*. It is apparent that, in the low-frequency regime, the effective shear and pressure wave speeds increase with *ε*, since the system becomes stiffer as *ε* is increased. At higher frequencies, the optical branches lift up as the stiffness of the internal links is increased. Moreover, for *ε*≃0.52 the highest finite stop-band becomes a partial stop-band, as shown in figure 9*d*, so that waves can propagate in some directions and are evanescent in the other directions. When *ε* → 1, two pairs of dispersion surfaces become closer to each other and they eventually coincide when *ε* = 1, forming a Dirac point. Figure 9*f* represents the dispersion diagram for a homogeneous triangular lattice (see, for instance, [32]).

### (c) Uni-directional waveforms in the heterogeneous triangular lattice

Now, we show that interfacial waveforms with preferential directionality can also be obtained in the heterogeneous triangular lattice by adjusting the gyroscopic spin directions. In the numerical simulations presented below, performed in Comsol Multiphysics, the lattice domain is a 90*l* × 76.2*l* rectangle. The size of the domain is smaller than that used for the hexagonal lattice, since the model for the triangular lattice presents a higher complexity and hence requires a larger computational cost due to the additional degrees of freedom. Apart from the different size and the additional links, the finite-element model developed for the triangular lattice is identical to that described in §2c.

**Figure 10.**
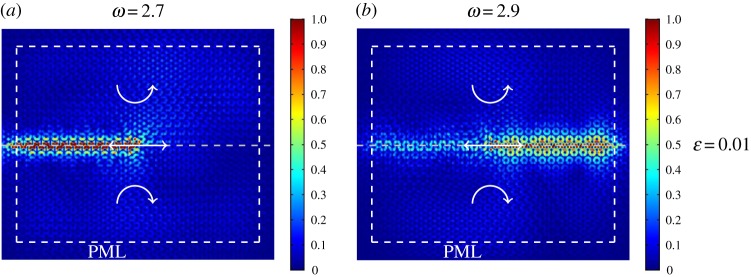
Elastic triangular lattice with soft internal links, whose domain is divided into two regions where the spinners rotate in opposite directions. A time-harmonic displacement is imposed on a node at the interface between the two regions. Interfacial waveforms are generated, whose direction depends on the frequency of the imposed displacement: (*a*) *ω* = 2.7, (*b*) *ω* = 2.9 (see also inset in figure 8*e*). In the computations, the absolute value of the spinner constant is |*α*| = 0.8 and the internal links inside the hexagonal cell are very soft (*ε* = 0.01). (Online version in colour.)

In figure 10, we divide the lattice domain in two regions, where the gyroscopic spinners are characterized by spinner constants of the same magnitude (|*α*| = 0.8) but of opposite sign. We apply a time-harmonic displacement to a lattice particle situated on the horizontal interface between the two regions. In part (*a*), the radian frequency is *ω* = 2.7, which is below the lower limit of the stop-band highlighted in figure 8*e*, corresponding to free vibrations in the analogous homogeneous infinite structure. Conversely, in part (*b*) the radian frequency of the external excitation is equal to *ω* = 2.9, which lies above the upper limit of the stop-band highlighted in figure 8*e*.

Comparing figure 10*a*,*b*, we observe that uni-directional interfacial waves can be created in the heterogeneous triangular lattice with soft internal links. This is due to the non-trivial topology of the band diagram, characterized by the presence of (almost formed) Dirac points. As in the illustrations for the hexagonal lattice studied in §2c and presented in figure 4*a*,*b*, the wave directionality in the heterogeneous triangular lattice also depends on the frequency of the external excitation. The thickness of the region in figure 10 where displacements are not zero is similar to that in figure 4, although it seems larger due to the difference in size of the computational windows. We have verified that for the triangular lattice the direction of wave propagation can also be reversed by changing the spin directions of the gyroscopic spinners.

**Figure 11.**
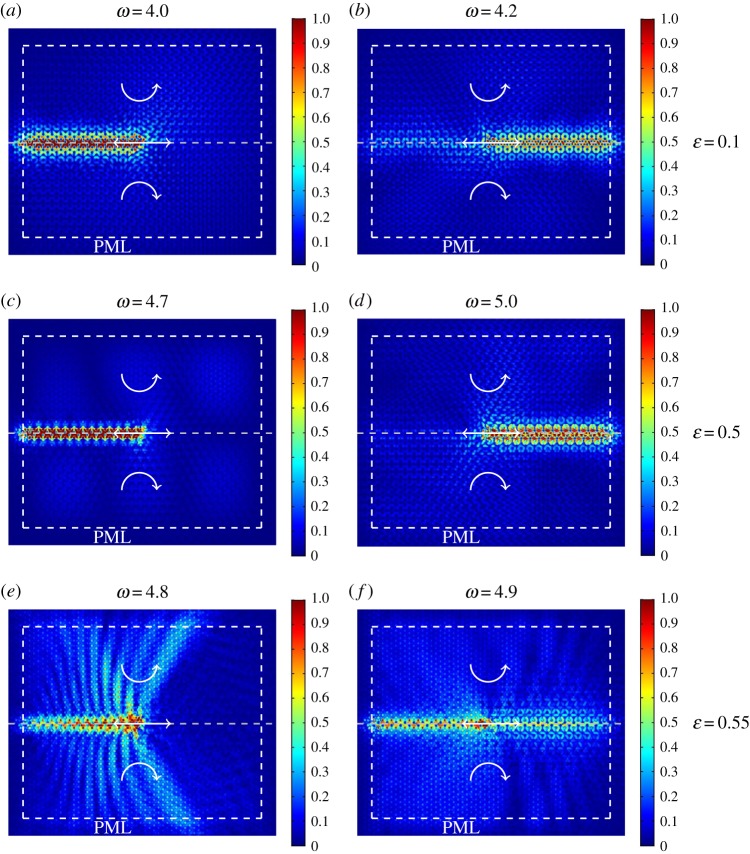
Computations showing the response of triangular lattices with internal links of different stiffness. Each lattice is attached to an inhomogeneous array of spinners. The computational set-up is described in the caption of figure 10, but in this case the absolute value of the spinner constant is |*α*| = 0.9. We consider three different values of the ratio *ε* of the stiffnesses of the links in the elementary cell of the lattice, and for each *ε* we take two frequencies, one above and one below the stop-band highlighted in figure 9. For *ε* = 0.1, the frequencies are *ω* = 4.0 (part *a*) and *ω* = 4.2 (part *b*); for *ε* = 0.5, *ω* = 4.7 (part *c*) and *ω* = 5.0 (part *d*); finally, for *ε* = 0.55, *ω* = 4.8 (part *e*) and *ω* = 4.9 (part *f*). (Online version in colour.)

Figure 11 shows the same lattice structure as in figure 10, but for |*α*| = 0.9. The stiffnesses of the internal links considered in figure 11*a*–*f* are larger than the value chosen in figure 10*a*,*b*. Nonetheless, if the system exhibits a stop-band near the (almost formed) Dirac point, in particular for *ε* = 0.1 and *ε* = 0.5 (figure 9*a*,*c*), uni-directional wave propagation can be realized in the triangular lattice (figure 11*a*–*d*). On the other hand, when the stop-band in the vicinity of the (almost formed) Dirac point is only partial (figure 9*d*), waves can also propagate in the bulk of the lattice (figure 11*e*,*f* ).

**Figure 12.**
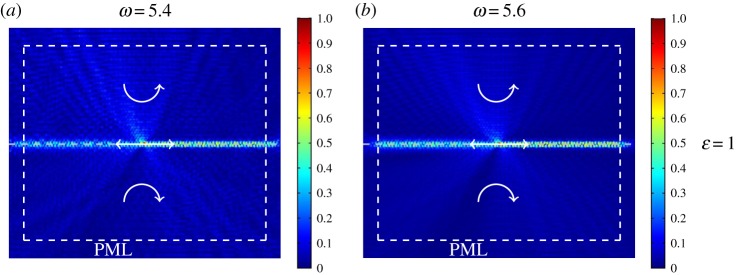
Displacement field in the homogeneous triangular lattice (*ε* = 1) produced by a time-harmonic displacement, having a frequency (*a*) lower and (*b*) higher than the frequency at the Dirac point (figure 9*f* ). In both cases, the absolute value of the spinner constant is |*α*| = 0.9. (Online version in colour.)

Now, we investigate the limit case when *ε* = 1, that is when the triangular lattice is homogeneous. The corresponding dispersion diagram, plotted in figure 9*f* , does not exhibit any finite width stop-bands, and it shows the formation of a Dirac point. The response of the homogeneous triangular lattice under a time-harmonic displacement is shown in figure 12*a*,*b* for the frequency *ω* = 5.4 (below the Dirac point) and *ω* = 5.6 (above the Dirac point), respectively. Wave localization is observed on the interface between the two media where spinners rotate in opposite directions, but without preferential directionality. In addition, waves of small amplitude propagate within the bulk. These results demonstrate that preferential directionality can be realized if the system exhibits a total stop-band near the Dirac point.

## 4. Conclusion

We have developed a novel approach to create uni-directional waveforms in discrete elastic periodic media without perturbing the structured system. This has been achieved by considering a hexagonal system of masses connected by elastic trusses and by the introduction of gyroscopic spinners, which are linked to individual nodes of the structure.

In the static regime, such a structure is statically under-constrained. In the time-harmonic regime, comprehensive dispersion analysis of a hexagonal lattice connected to gyroscopic spinners has been presented and special features of the dispersive behaviour have been identified. In particular, we have shown that using gyroscopic spinners provides an effective tool for manipulating stop-bands in the vicinity of Dirac cones. These chiral structures have been used to create uni-directional interfacial waveforms with a tunable direction. To the best of our knowledge, the present paper shows for the first time inhomogeneous elastic chiral structured media which exhibit interfacial waveforms with preferential directionality.

Numerical illustrative examples have been used to demonstrate this phenomenon for a variety of configurations. In particular, it has been shown that the directionality of the interfacial waveforms can be changed by reversing the spin directions of the gyroscopic spinners or by changing the frequency of the excitation.

The hexagonal elastic lattice represents the limit case of a triangular lattice with very soft internal links within its hexagonal cells. Indeed, when gyroscopic spinners are attached to the triangular lattice, interfacial leaky waves can be generated at the boundaries of regions where spinners rotate in opposite directions. The effect of preferential uni-directionality is absent when the triangular lattice consists of uniformly distributed elastic rods of equal stiffness, as demonstrated in figure 12.

The designs proposed here open new ways of generating and controlling topologically protected states in elastic structures through the use of chiral metamaterials.

## Supplementary Material

Video 1

## Supplementary Material

Video 2

## Supplementary Material

Supplementary Material
