# Supplementary material for: Interfacial waveforms in chiral lattices with gyroscopic spinners
Source: Proc Math Phys Eng Sci. 2018 Jul 25;474(2215):20180132. doi: 10.1098/rspa.2018.0132 (PMC6083240; doi:10.1098/rspa.2018.0132)
Supplement: Supplementary Material [file rspa20180132supp3.pdf]

Supplementary Material

Title: Interfacial waveforms in chiral lattices with gyroscopic spinners

Authors: M. Garau[1], G. Carta[2], M.J. Nieves[3,1,*], I.S. Jones[2], N.V. Movchan[4], A.B. Movchan[4]

[1] Keele University, School of Computing and Mathematics, Keele, ST5 5BG, UK
[2] Liverpool John Moores University, Mechanical Engineering and Materials Research Centre, Liverpool, L3 3AF, UK
[3] University of Cagliari, Department of Mechanical, Chemical and Material Engineering, Cagliari, 09123, Italy
[4] University of Liverpool, Department of Mathematical Sciences, Liverpool, L69 7ZL, UK
[*] Corresponding author
Email address of the corresponding author: m.nieves@keele.ac.uk

Determination of the spinner constant α

Each particle of the lattice is connected to a gyroscopic spinner, shown in Fig. S1. The gyroscopic spinner is pinned at the bottom end, where it can rotate but cannot translate. We denote by ψ, ϕ and θ the angles of spin, precession and nutation, respectively.

The equations of motion of the gyroscopic spinner are given by [1]

$$M_{x'} = I_0\ddot{\theta} + (I - I_0)\dot{\phi}^2 \sin(\theta)\cos(\theta) + I\dot{\phi}\dot{\psi}\sin(\theta)\,, \tag{S1a}$$

$$M_{y'} = I_0\ddot{\phi}\sin(\theta) + (2I_0 - I)\dot{\phi}\dot{\theta}\cos(\theta) - I\dot{\theta}\dot{\psi}\,, \tag{S1b}$$

$$M_{z'} = M_z = I\left[\ddot{\phi}\cos(\theta) - \dot{\phi}\dot{\theta}\sin(\theta) + \ddot{\psi}\right]\,. \tag{S1c}$$

Since the displacement of the lattice particle is small, the nutation angle of the gyroscopic spinner is also small, namely $|\theta| \ll 1$. Accordingly, the equations of

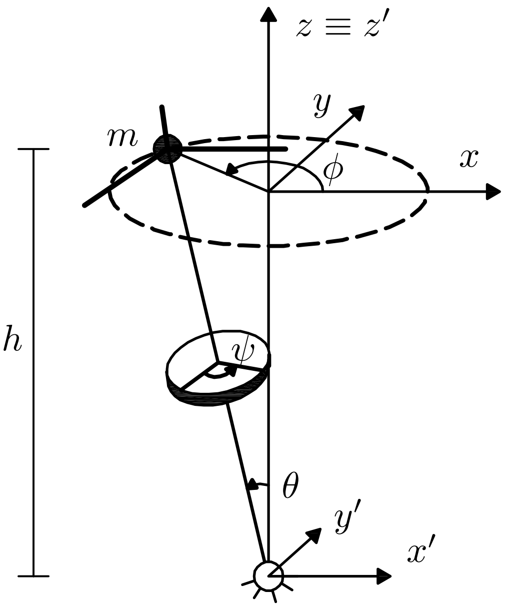

Figure S1: Schematic representation of a gyroscopic spinner.

motion (S1) to leading order take the form:

$$M_{x'} = I_0\ddot{\theta} + (I - I_0)\dot{\phi}^2\theta + I\dot{\phi}\dot{\psi}\theta\,, \tag{S2a}$$

$$M_{y'} = I_0\ddot{\phi}\theta + (2I_0 - I)\dot{\phi}\dot{\theta} - I\dot{\theta}\dot{\psi}\,, \tag{S2b}$$

$$M_{z'} = M_z = I\left(\ddot{\phi} - \dot{\phi}\dot{\theta}\theta + \ddot{\psi}\right)\,. \tag{S2c}$$

We assume that the spin and precession rates are constant, i.e. $\dot{\psi} = \text{Const} = \Omega$ and $\dot{\phi} = \text{Const}$. Furthermore, neglecting the effect of gravity, $M_{x'} = M_{y'} = 0$. Hence, we obtain:

$$0 = I_0\ddot{\theta} + (I - I_0)\dot{\phi}^2\theta + I\Omega\dot{\phi}\theta\,, \tag{S3a}$$

$$0 = (2I_0 - I)\dot{\phi}\dot{\theta} - I\Omega\dot{\theta}\,, \tag{S3b}$$

$$M_{z'} = M_z = -I\dot{\phi}\dot{\theta}\theta\,. \tag{S3c}$$

Here, Eq. (S3b) leads to

$$\dot{\phi} = \frac{I}{2I_0 - I}\Omega\,. \tag{S4}$$

In the time-harmonic regime, the nutation angle has the form $\theta = \Theta e^{i\omega t}$, where ω is the radian frequency of the lattice. Substituting this form into Eq. (S3a) and

using Eq. (S4), we determine the following *compatibility condition* between the spin rate Ω and the radian frequency ω:

$$\Omega = \pm \frac{2I_0 - I}{I} \omega \, . \tag{S5}$$

Comparing Eqs. (S4) and (S5), we observe that $\dot{\phi} = \pm\omega$.

Finally, using Eq. (S3c), we derive the expression for the moment M_z imposed by the gyroscopic spinner on the lattice particle attached to it:

$$M_z = \mp \mathrm{i} \omega^2 \theta^2 \, . \tag{S6}$$

In the linearised case, the nutation angle is given by $\theta = |\boldsymbol{u}| / h$, where $|\boldsymbol{u}|$ is the magnitude of the (in-plane) particle displacement and h is the height of the spinner (see Fig. S1). Hence, the force applied to the lattice particle by the gyroscopic spinner is

$$F = \frac{M_z}{|\boldsymbol{u}|} = \mp \mathrm{i} \frac{I}{h^2} \omega^2 \, |\boldsymbol{u}| \; . \tag{S7}$$

Consequently, the spinner constant appearing in Eqs. (2.1) and (3.1) of the main text is given by

$$\alpha = \frac{I}{h^2} \, , \tag{S8}$$

as also shown in [2].

References

[1] Goldstein H, Poole C, Safko J. 2002 *Classical Mechanics*, 3rd edition. San Francisco: Addison Wesley.

[2] Brun M, Jones IS, Movchan AB. 2012 Vortex-type elastic structured media and dynamic shielding. *Proc. R. Soc. A* **468**, 3027–3046. (doi: 10.1098/rspa.2012.0165)
